# The Use of Newer High Translucency Zirconia in Aesthetic Zone

**DOI:** 10.1155/2014/432714

**Published:** 2014-03-04

**Authors:** Zishan Dangra, Mahesh Gandhewar

**Affiliations:** ^1^Private Practice, Mumbai 400703, India; ^2^Department of Prosthodontics, ACPM Dental College, Dhule 424001, India

## Abstract

Loss of anterior tooth causes aesthetic and functional disharmony. Although no restorative material can approach the appearance of intact tooth enamel, glass ceramic, at the increased risk of brittle fracture, can mimic original tooth color better than the other restorative options. The newest zirconia material comes with unparalleled individualization in aesthetics and optimal physical properties. One of the basic principles of tooth preparation is conservation of tooth structure. This clinical report describes the replacement of maxillary and mandibular incisor with latest generation zirconia adhesive fixed partial denture. The authors have achieved unmatched aesthetics with newer high translucency zirconia.

## 1. Article Proper

In the field of indirect restorative materials, it has always been desirable to have the right blend of aesthetics and durability. The zirconia material conquers all domains of dental restorations where extreme precision, strength, and aesthetics are required. The recently introduced high performance zirconia adds to its appearance by having improved translucency, that is, although inferior to glass ceramic but of course without as much as two thirds reduction in flexure strength.

With respect to dental restoration, aesthetics means color that is matched perfectly in lightness to its adjacent teeth and translucency. Translucency refers to having the appearance between complete opacity and complete transparency. In order to produce lifelike restoration, the light must be transmitted and reflected as it does with the natural tooth and it's done.

Lava plus high translucency zirconia is recently introduced by 3M ESPE (3M ESPE, St Paul, MN). It is an after comer of original Lava zirconia, which is considered as one of the most successful ceramic systems. The newer version can be fabricated with hand layered, pressed, or monolithic options. This material is available with the widest range of dyeing liquids and Lava plus effect shades for excellent color match with VITA classical shade guide and unprecedented customization. It is claimed to have the only zirconia system with direct conversion to VITA system 3D-MASTER shades. The translucency of zirconia material is determined by impurities and structural defects as it causes light absorption and scattering, respectively. The high quality processing of Lava plus minimizes the effects of impurities and structural defects. The translucency of zirconia material is reduced by different refractory index and segregation nature of the alumina which is added into zirconia for aging stability. In Lava plus zirconia, the alumina content is reduced to 0.1 wt% and distribution is improved while maintaining the aging stability of the material [1, 2].

The strength of new zirconia material is no way inferior to Lava zirconia. The high strength of the material allows thinner walled restorations to add to the translucency and conserves the tooth structure, of course.

The anterior restorations in the following case illustrate the use of this material to achieve highly aesthetic result.

## 2. Clinical Report 

**Figure 1:**
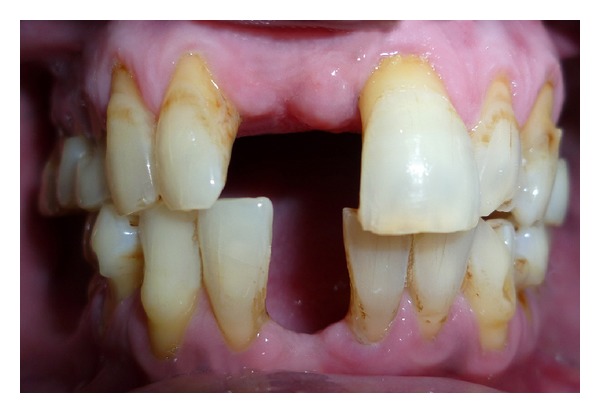
Frontal pretreatment view.

**Figure 2:**
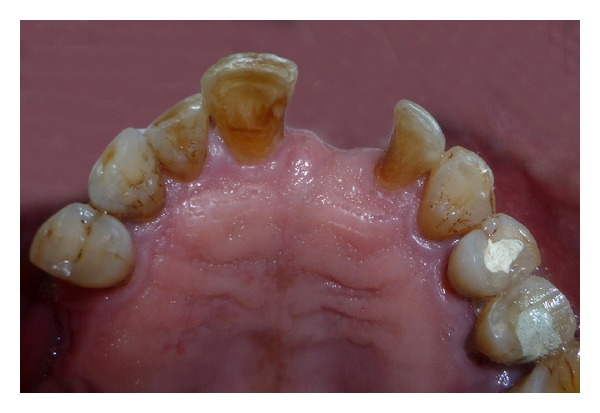
Abutment maxillary tooth preparation.

**Figure 3:**
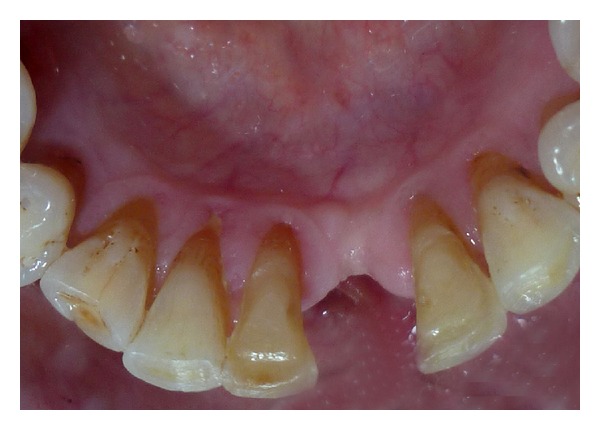
Abutment mandibular tooth preparation.

A 52-year-old man was diagnosed with partial anodontia with missing maxillary and mandibular right central incisor (Figure 1). Various prosthetic treatments were suggested; all ceramic adhesive fixed partial denture (FPD) was accepted by the patient. Apart from the conventional FPD, an implant supported single crown is considered viable treatment alternative to resin bonded FPD [3]. Although implant supported crown is noninvasive to teeth adjacent to edentulous area(s), it may not be advisable due to anatomic limitations and economic constraints. Metal-ceramic resin bonded FPDs have disadvantage of grayish hue on incisal third of abutments due to the cast metal retainers [4, 5]. For zirconium resin bonded FPDs, patient selection is limited to those with sound abutments with minimal or no restorations and stable occlusion [6]. Before proceeding with actual tooth preparation, diagnostic waxing was performed on diagnostic casts and presented to the patient. Tooth preparation was made by flame shaped and round end shoulder diamond points. The lingual and palatal surfaces of the abutment teeth were reduced to 0.5 mm. Shoulder finish line with rounded internal angle and 5′ degrees horizontal divergence extends supragingivally. The proximal reduction of the preparation should end just lingual to the labioproximal line angle on the edentulous side and on the dentulous side, as far as access permits [7]. The proximal grooves were prepared on the edentulous side with flat end tapered diamond to increase retention of the final prosthesis [8] (Figures 2 and 3). Gingival retraction was not carried out as preparation margins were supragingival. An impression was made with vinyl polysiloxane heavy body/light body impression material (Imprint 3, 3M ESPE, St Paul, MN). Final shade was taken for the prostheses. Provisional restorations were fabricated using diagnostic wax-up and Bis-acrylic composite temporization material (Protemp Plus, 3M ESPE, St Paul, MN).

**Figure 4:**
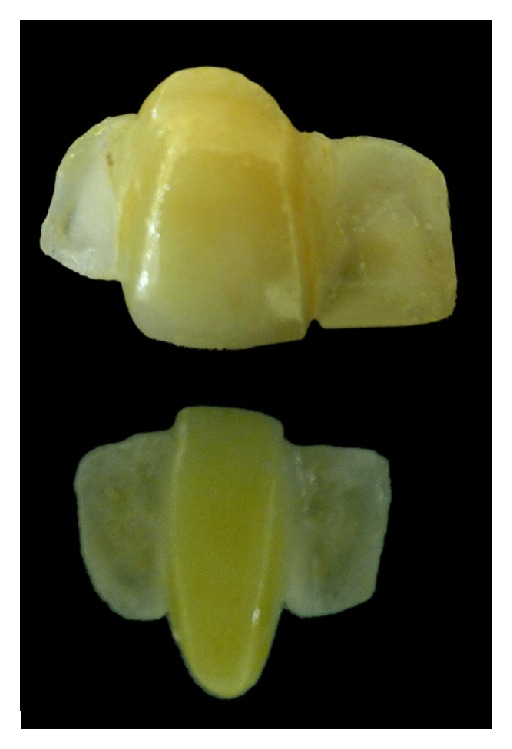
Definitive restorations.

In the next appointment, the provisionals were carefully removed. The Lava plus zirconia frameworks fabricated with CAD/CAM were evaluated intraorally for marginal fit, occlusion, and so forth. Subsequently, traditional hand layered technique was applied to allow restorations that exactly mimic the nature (Figure 4).

**Figure 5:**
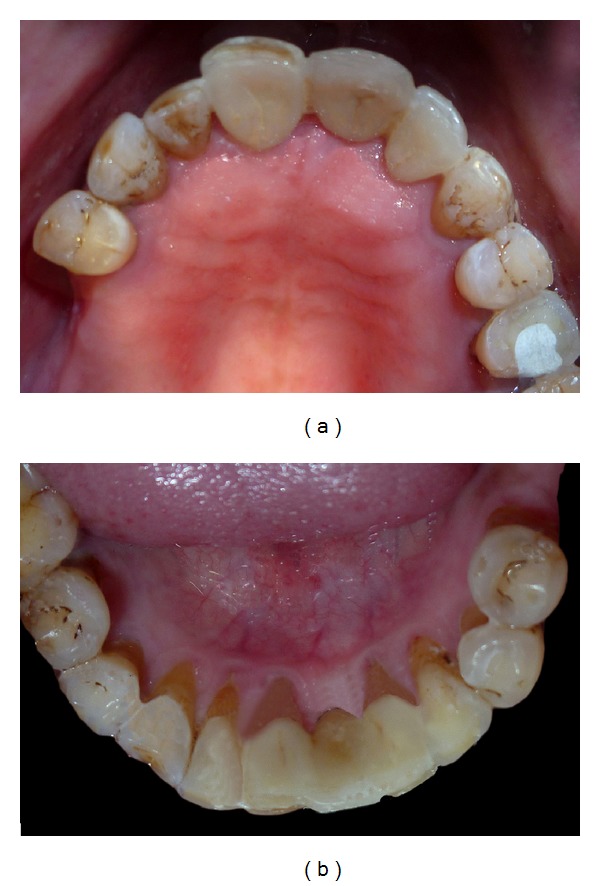
Lingual view of cemented restorations.

**Figure 6:**
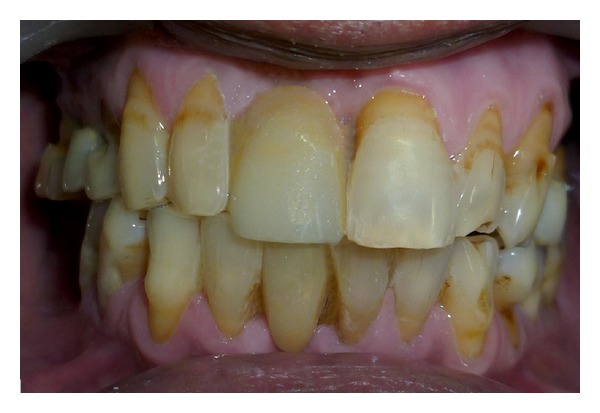
Frontal view of cemented restorations.

The intaglio surface of the prostheses was grit blasted with <50 *μ*m aluminium oxide grain and treated with alloy primer. The prepared enamel surfaces were isolated, cleaned with prophylaxis paste, rinsed with water, air-dried, and etched with 37% phosphoric acid for 20 seconds. The dual cure adhesive resin cement (Panavia Fluoro; Kuraray Medical Inc.) was used for luting the restorations to the abutment teeth. Excess cement was removed by hand waving technique, after brief tack light cure for 1 second, while holding the restoration in place. Restorations were light- cured according to manufacturer's recommendations for 20 seconds from all possible aspects (Figures 5 and 6). The patient was examined twice in a preceding year. The Lava plus zirconia adhesive FPDs were found satisfactory in aesthetics.

## 3. Discussion

High translucency zirconia is an option worth considering for restorations that need to be aesthetically superior and serve patient well for years. For Lava plus anterior adhesive bridge, the selected case should not have deep bite, heavy bruxism, and mobile abutments. The clinical protocol described in this article is straightforward and exactly the same as recommended. Considering laboratory protocol, the recommended connector cross section is 7 mm^2^. Veneering of Lava plus zirconia restoration has three options as discussed above. The Lava plus monolithic veneering technique is selected when the clinical situation demands high strength or when there is reduced interocclusal space. The technique for bonding the restoration is essentially the same as with any other zirconia material. However, conventional cements can be used with traditional anterior FPD made of high translucency zirconia which is an added advantage over glass ceramics.

## 4. Summary 

This clinical report illustrates a conservative treatment with Lava plus zirconia adhesive FPD for the replacement of maxillary and mandibular incisor. The mechanism behind optimized translucency and color is described with case selection, specific tooth preparation steps, and clinical procedure involved in the newer high translucency zirconia adhesive FPD.
